# Treatment of porcine ovarian follicles with tert-butyl hydroperoxide as an ovarian senescence model *in vitro*

**DOI:** 10.18632/aging.204831

**Published:** 2023-07-05

**Authors:** Peihua Shi, Jinchun Gao, Shunran Zhao, Wei Xia, Junjie Li, Chenyu Tao

**Affiliations:** 1 College of Animal Science and Technology, Hebei Agricultural University, Baoding, Hebei, China

**Keywords:** antral follicles, porcine, oxidative stress, aging, t-BHP

## Abstract

Ovarian aging is the main reason of female reproductive problems. Excessive oxidative stress can induce ovarian senescence and follicular atresia, thereby reducing the reproductive performance. Follicles were divided into five groups for *in vitro* culture based on the duration of stimulation with tert-butyl hydroperoxide (t-BHP)—control group and groups 1 h, 2 h, 6 h, and 12 h. The results revealed that the ratio of progesterone (P_4_) to estradiol (E_2_) was increased after 24 and 36 h of follicle culture, shifting follicles toward atresia (*P* < 0.05). Stimulated by 200 μM t-BHP, follicles showed progressive aging phenotype. Senescence-associated β-galactosidase staining (SA-β-Gal) showed a significant increase in the number of positive cells (*P* < 0.05). Reactive oxygen species were also significantly upregulated (*P* < 0.05). t-BHP treatment for 6 h induced significant increases in Caspase 3, P53, and Foxo1 mRNA and protein levels (*P* < 0.05) and significant decreases in SOD mRNA and protein levels (*P* < 0.05). Transcriptome sequencing analysis of the follicles showed that the aged and treatment groups were clustered together in hierarchical clustering. Correlation analysis indicated significant changes at the transcriptome level in the treatment groups versus the control group. The common differentially expressed genes in the treatment groups were enriched in three growth-factor signaling pathways associated with cell proliferation and apoptosis (P53, mTOR, and MAPK). In conclusion, induction of follicular senescence by treatment with 200 μM t-BHP for 6 h is an effective *in vitro* model to simulate ovarian senescence in sows.

## INTRODUCTION

Aging is often accompanied by a disruption of homeostasis and a decline in the ability of cells to clear out damaged cellular components through the process of autophagy. This decline is primarily caused by dysfunction of proteins and mitochondria, which in turn leads to chronic inflammation and ultimately, sterile inflammation [1, 2]. Cellular senescence is a state induced by stress and certain physiological processes and is characterized by hormone secretion, DNA damage, oxidative stress, prolonged cell cycle of metabolic changes, and usually irreversible cell-cycle termination [3]. As cells divide, they have a limited ability to replicate, known as the Hayflick limit. Once this threshold is reached, the cells are no longer able to divide, even with increased stimulation. If a wide variety of proliferating cells in a tissue undergo cell senescence, the regenerative ability of the tissue is reduced. Cellular senescence is commonly associated with aging, leading to a decreased regenerative ability, which serves as a hallmark of tissue senescence [4]. Accumulation of senescent cells is a common occurrence with age across various tissues [5–7].

In female mammals, aging has an extensive impact on reproductive performance. Ovarian senescence is a natural process that results in a decline in the ability of the ovaries to produce fully functional oocytes. It is characterized by the continuous loss of follicles and a reduction in the overall number of follicles, which ultimately leads to a gradual decline in female fertility. Furthermore, due to a decrease in ovarian follicle cells, ovarian aging leads to the failure of the ovary to produce adequate sex hormones, which can impact the maintenance of the physiological functions in animals or humans [8, 9]. The gradual decline in the number of ovarian follicles with age results in hormonal imbalances and irregularities in the ovarian cycle. Furthermore, aging ovaries produce poor-quality oocytes, which can cause developmental stagnation, aneuploidy, implantation failure, and abortion [10–12]. Granulosa cells and corpus luteum cells in the follicles are essential sources of estrogen and progesterone for female physiological functions. Prolonged arrest of granulosa cells can inhibit the ability of ovarian follicles to mature, ultimately leading to atresia of immature follicles.

As sows age, their reproductive physiology undergoes significant changes. Fecundity decreases while the elimination rate increases, leading to a decrease in dominant sows and a serious loss in production efficiency. The ovaries of pigs typically develop between 72 and 165 d of age. HE staining for atresia regulation of follicles in the ovarian tissues of pigs confirmed that during oocyte proliferation, spare oocytes underwent apoptosis. At the follicular stage, atresia of a wide range of primordial and dominant follicles was observed, and the total primordial follicle population showed constant atresia [13]. Atresia of the follicles at all stages, including primordial and dominant follicles, can be observed during the fast follicular growth phase. However, the degeneration of primordial follicles appears to be the most significant. TUNEL staining has been used in some studies to examine the atresia of a large variety of follicles in the oocyte proliferation stage, and it has been confirmed that atresia is caused by the apoptosis of follicular oocytes [14]. Atresia can occur at all stages of the follicle development. The direct reason of follicle atresia is the apoptosis of follicular granulosa cells. Apoptosis of granulosa cells was once thought to be the main cause of atresia in secondary follicles. However, as follicles continue to grow and proliferate rapidly, atresia at all stages becomes increasingly evident.

It has been reported that chemotherapeutics, including cyclophosphamide, cisplatin, Tripterygium Wilford, and hydrocortisone, can negatively impact ovarian function and lead to reproductive issues. The frequent use of chemotherapy to manage diseases has resulted in an increased incidence of these complications. Cyclophosphamide is particularly toxic to the ovary. Its mechanism of action involves inhibiting the synthesis of DNA, RNA, and protein, leading to gonadal damage [15]. Because the technology of ovarian culture is limited and most of the chemical methods are based on the mouse [16], there are few aging models of porcine ovaries. Most *in vitro* aging models are constructed using cells, and oxidative damage is one of the most commonly used methods to construct aging models. Various cells have been subjected to oxidative stress-inducing conditions for experimental purposes. For example, human skeletal myoblasts were treated with 1 mmol/L hydrogen peroxide for 30 min to investigate the regulatory effect of the tocotrienol-rich fraction on senescence [17]. Another study has reported that t-BHP can induce the aging of hematopoietic stem cells in mice, and the proportion of aging cells was > 50% after 6 h [18].

It is crucial to establish an *in vitro* aging model to study the biological functions of ovarian aging, as it can be challenging to obtain samples from high-parity sows. In this study, an *in vitro* aging model of follicles was established, providing valuable experimental data for investigating the mechanisms of delaying aging on reproductive physiology.

## RESULTS

### Quality characterization of porcine antral follicles

**Figure 1:**
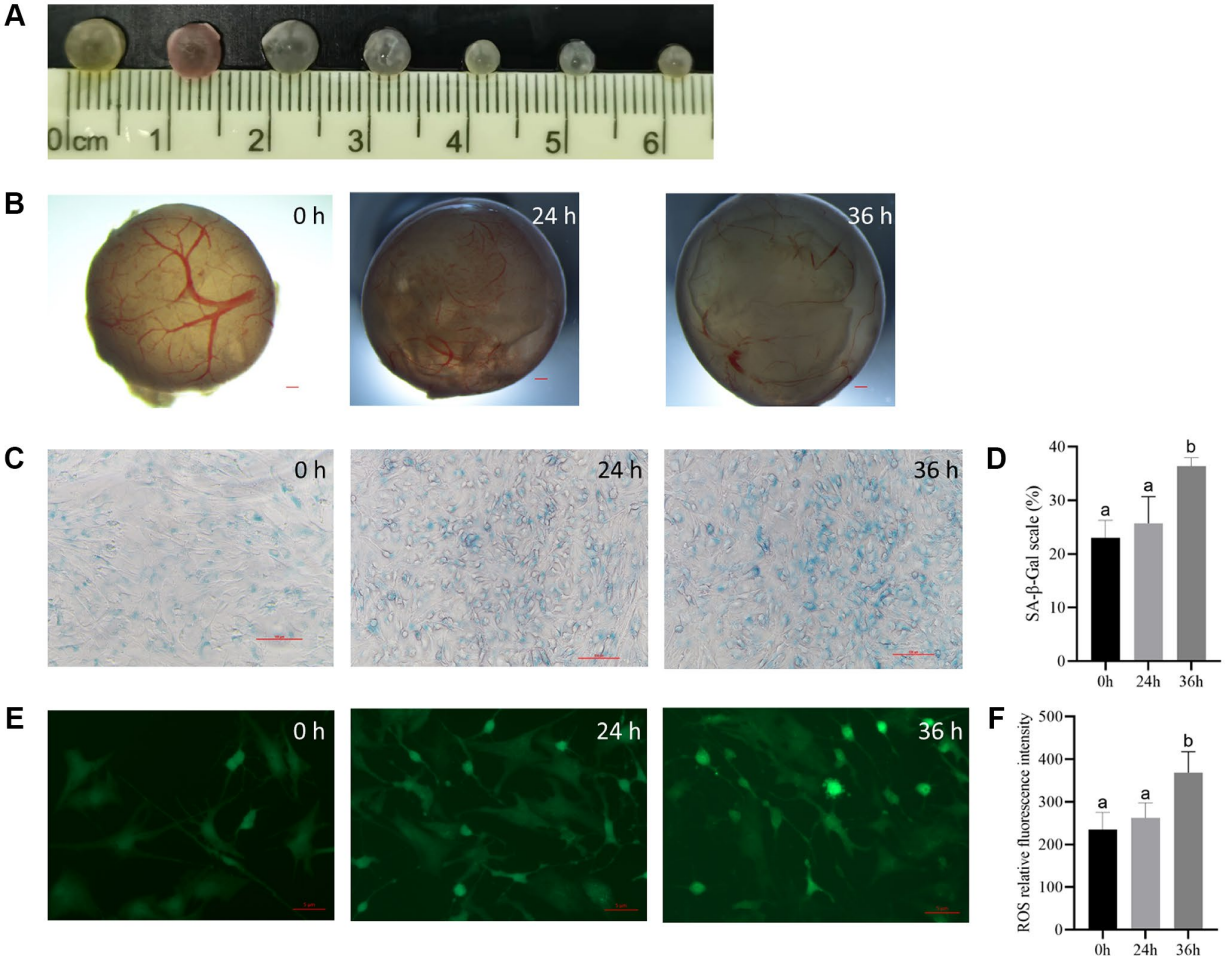
**The culturing system of porcine antral follicles is feasible within 24 h.** (**A**) The antral follicles with a diameter of 3–5 mm was selected. (**B**) The antral follicles were cultured *in vitro* for 24 and 36 h. Bar, 300 μm. (**C**) SA-β-Gal staining of the granulosa cells. Bar, 100 μM. (**D**) Statistics of positive cells at different time points. *P* < 0.05. (**E**) ROS fluorescence intensity staining of follicles in each group. Bar, 5 μm. (**F**) Relative ROS levels. *P* < 0.05.

According to size (3–5 mm in diameter), healthy follicles were selected (Figure 1A), three groups of follicles were cultured and observed under a microscope to judge the morphology of isolated porcine follicles. The morphological characteristics of the follicles at each stage were observed and recorded as follows: The control group exhibited an intact, even, and compact follicle wall, with some pink or yellow regions, and well-distributed, bright red capillaries. The follicular fluid was clear, and there were no shed granulosa cells present in the follicular cavity. In the 24-h group, the outer layer of the follicle wall was intact, but the basement membrane was partially degraded, and the capillary network was decreased to varying degrees. The follicular fluid was clear, and the follicular cavity was clean without any lumps. In the 36-h group, the outer layer of the follicle wall was still intact, but fewer capillaries were observed. A few granulosa cells exfoliated into the follicular cavity, and small flocculent deposits appeared in the cytoplasm (Figure 1B). The granulosa cells in the parietal layer of the follicles were stained for SA-β-Gal to observe cellular senescence. The results showed that the cells in the control group were small, round, and closely connected. In contrast, the positive cells in treatment group were highly stained and showed uneven morphology, significant differentiation, large volume, and loose radial connections (Figure 1C), and the proportion of positive cells was found to not be significantly higher in the treatment group at 24 h and 36 h than in the control group (Figure 1D).

Results from fluorescence staining showed that although the level of reactive oxygen species (ROS) was not significantly higher in the 24 h group than in the control group, the difference was significant at 36 h (Figure 1E and 1F).

### Changes in the progesterone and estrogen levels in follicles

Progesterone can be synthesized in granulosa and membrane cells of follicles, and a low concentration of progesterone contributes to the formation of the luteinizing hormone peak, which induces ovulation in mature follicles. Estrogen stimulates granulosa cell proliferation and prevents apoptosis. Changes in hormone levels can reflect the degree of follicular atresia. The hormone level measurement results indicated a significant decrease in E2 concentration and a significant increase in P4 concentration after 36 h, while no significant difference was observed between the two groups at 24 h. The P4/E2 ratio also showed a significant increase with an increase in follicular culture duration (Table 1). These findings suggest that atresia did not occur in the follicles during *in vitro* culture. Based on these results, 24 h culture of follicles was concluded to be suitable for following experiments.

**Table 1:** The changes of hormone level in follicles.

**Processing time**	**Progesterone concentration (pmol/L)**	**Estradiol concentration (pmol/L)**	**P_4_/E_2_**
0 h	140.00 ± 19.51^a^	218.70 ± 4.94^a^	0.64 ± 0.08^a^
24 h	190.00 ± 3.21^b^	198.52 ± 7.04^b^	0.96 ± 0.02^b^
36 h	223.33 ± 14.70^b^	149.58 ± 4.26^c^	1.50 ± 0.13^c^

Data are represented as mean +/− SEM, different letters indicate statistically significant differences (*P* < 0.05).

### The optimal duration of t-BHP treatment to induce follicular senescence

We tested whether treatment with 200 μM t-BHP for 1, 2, 6, or 12 h induced follicular senescence. After the treatment, the follicles were cultured in drug-free medium for 24 h, and granulosa cells were collected and cultured until they adhered to the substratum.

**Figure 2:**
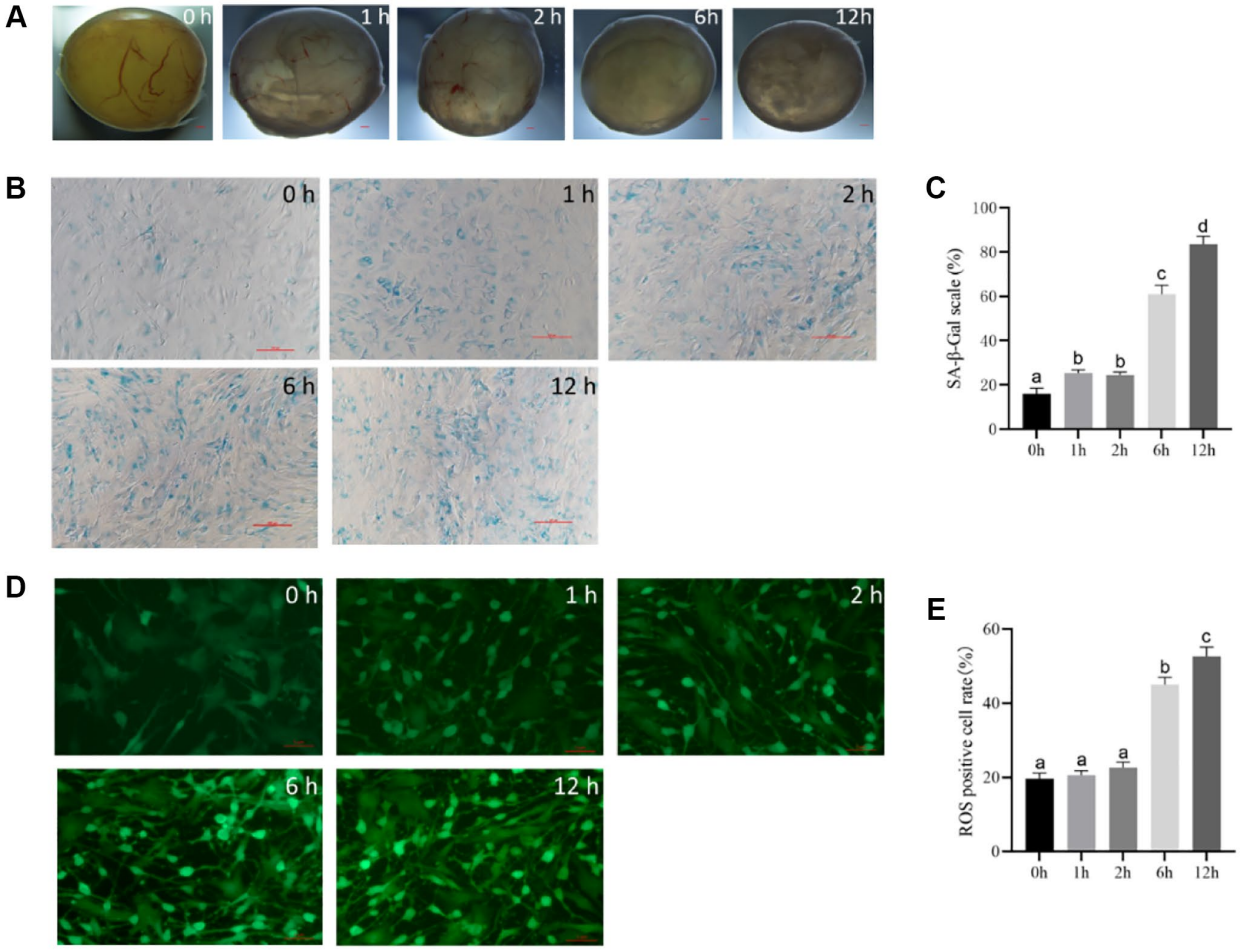
**The optimal time of follicular senescence induced by t-BHP.** (**A**) Treating with 200 μM t-BHP, and culturing antral follicles *in vitro* for 1 h, 2 h, 6 h, and 12 h. Bar, 300 μm. (**B**) SA-β-Gal staining of granulosa cells at different time points with 200 μM t-BHP treatment. Bar, 100 μm. (**C**) Statistics of positive cells after different time of t-BHP treatment. *P* < 0.05 (**D**) ROS fluorescence intensity staining in follicles of each group treated with 200 μM t-BHP at different times. Bar, 5 μm. (**E**) Relative ROS levels after different t-BHP treatment times *P* < 0.05.

The follicular phenotype showed intact follicular walls, visible blood vessels, and a clear follicular cavity 1 h and 2 h after culture (Figure 2A). There were fewer follicular vessels in the 6 h group than in the control group, and a small number of granular cells clumps appeared in the follicular cavity (Figure 2A). However, at 12 h, the blood vessels in the wall of the follicles disappeared, and the color of the follicles gradually became gray, with flocculent deposits in the lumen. The granulosa cells in the parietal layer broke off into clumps, unlike the cells in the control group (Figure 2A).

SA-β-Gal staining of the follicular granulosa cells in each group revealed no significant changes in the 1 h and 2 h groups, with a small percentage of cells showing blue staining and a positive-cell proportion of <40%. In the 6 h group, the positive cells were looser and larger compared with those in the control group, and the positive staining rate was up to 60% in five randomly selected fields, indicating partial cellular senescence. In the 12 h group, the cells showed distinct morphological changes, such as polygonal shape, dilation, and flattening, with a decreased refractive index, and the majority of the cells were senescent, with a positive staining rate of more than 80% in five random fields. These results suggest that t-BHP treatment at 200 μM for 6 h and 12 h can induce significant senescence in porcine follicular granulosa cells (Figure 2B and 2C).

ROS fluorescence staining revealed weak ROS staining in the control group, and staining was greater in the 1-h and 2-h groups, but there was no significant difference between the two groups. However, after 6 h and 12 h, the expression levels of ROS were significantly increased. There was a progressive increase in ROS expression over time, with strong fluorescence staining of granulosa cells and a poorer cell status observed in the 12-h group (Figure 2D and 2E). The shortest time for ROS to appear significantly elevated was 6 h. Therefore, the optimal time to induce follicular senescence under the experimental conditions was determined to be 6 h with 200 μM t-BHP.

### Effect of t-BHP on the gene expression levels in porcine follicles

**Figure 3:**
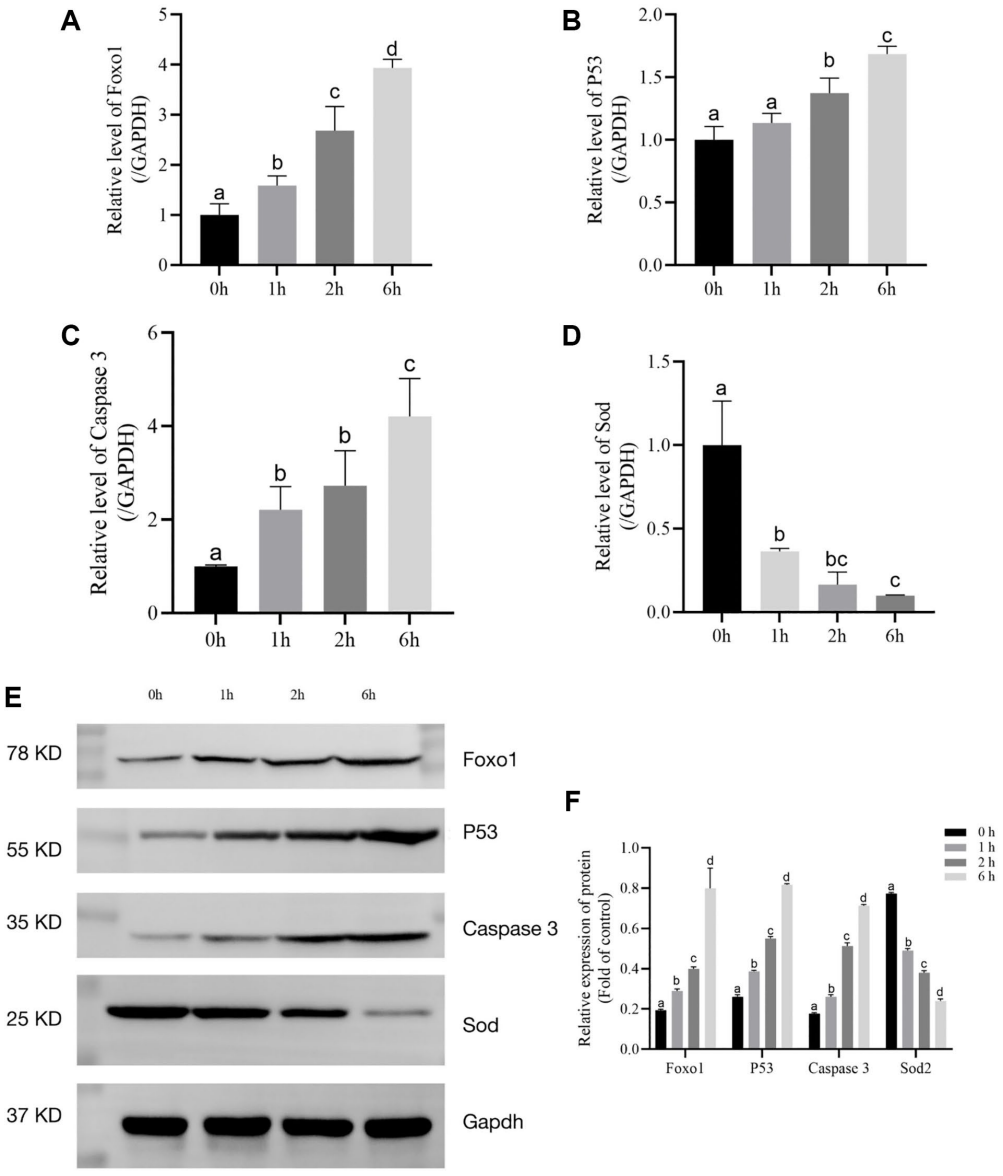
**The relative expression levels of *Foxo1*, *P53*, *Caspase 3*, and *SOD* after t-BHP treatment for 6 h.** (**A**) Relative expression of mRNA level of *Foxo1* in t-BHP- induced follicles. (*P* < 0.05). (**B**) Relative expression of mRNA level of *P53* in t-BHP- induced follicles. (*P* < 0.05). (**C**) Relative expression of mRNA level of *Caspase 3* in t-BHP- induced follicles. (*P* < 0.05). (**D**) Relative expression of mRNA level of *SOD* in t-BHP-induced follicles. (*P* < 0.05). (**E**) Relative protein expression levels of Foxo1, P53, Caspase 3, SOD2, and GAPDH in follicles. (**F**) Statistical analysis of Foxo1, P53, Caspase 3, and SOD2 in follicles. (*P* < 0.05).

*Foxo1*, *P53*, *Caspase 3*, and *SOD* mRNA levels were assessed via qRT-PCR. *Foxo1*, *P53*, and *Caspase 3* mRNA levels were markedly increased by t-BHP treatment. However, t-BHP decreased the level of *SOD* mRNA (Figure 3A–3D).

Foxo1, P53, Caspase 3, and SOD protein levels were assessed via western blot analysis. As shown in Figure 3E and 3F, t-BHP treatment upregulated P53, Caspase 3, and Foxo1, compared with the control levels (*P* < 0.05). We also found that t-BHP significantly downregulated SOD (*P* < 0.05). Taken together, these data implied that t-BHP (200 μM) treatment for 6 h could effectively accelerate senescence through Foxo1, P53, Caspase 3, and SOD2.

### Transcriptional landscape of porcine follicles

**Figure 4:**
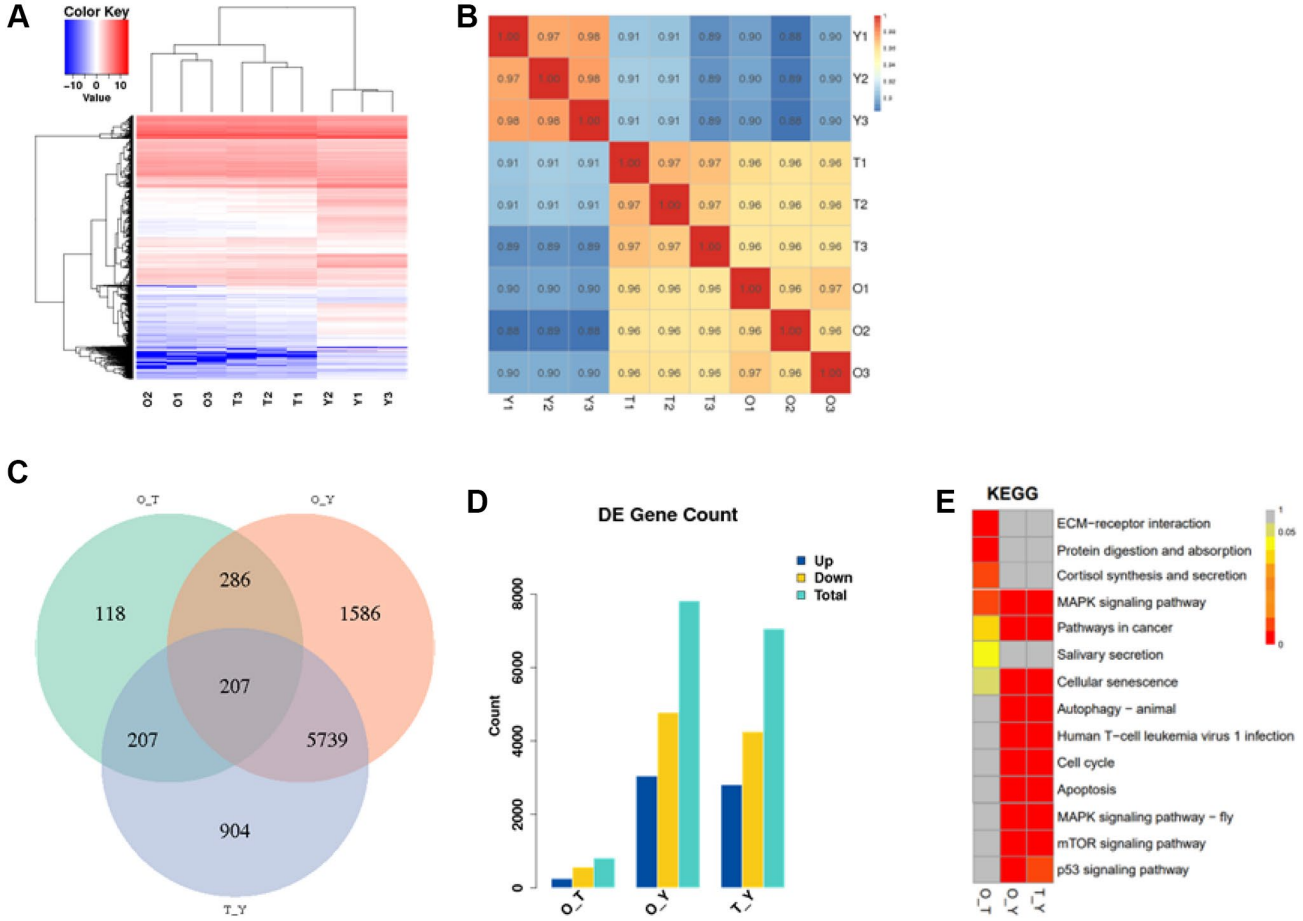
**The transcriptional landscape of porcine follicles.** (**A**) Heat map and hierarchical clustering of differentially expressed genes. Red color indicates high gene expression and blue color indicates low gene expression. (**B**) Correlation heatmap of transcriptomic similarity among 6 aging follicles samples and 3 young control follicles tissues. Inter-sample gene expression correlation is indicated by the color bar above the heatmap. (**C**) Venn diagram showing the intersection of DEGs among two normal control follicles vs. Treatment. DEGs were identified by the R package DESeq2 under the cutoff of adjusted *P* < 0.05. (**D**) Statistical graph of differentially expressed genes. (**E**) Distribution of differential gene enrichment pathways in each comparison group. Different colors represent different degrees of enrichment, the redder the color the more significant the enrichment. (Abbreviations: O: Old; T: Treatment; Y: Young).

We performed transcriptome sequencing (RNA-seq) on 6 groups of porcine follicle samples (3 groups of young and 3 groups of aged porcine follicle samples) and 3 follicle tissue in t-BHP treatment groups (Treatment) (Figure 4). Hierarchical clustering showed that groups of aged (O) and groups of t-BHP treatment (T) were classified together (Figure 4A). The similarity heatmap showed that aging model follicles displayed more pronounced transcriptomic changes (Figure 4B). A total of 207 common DEGs were identified in the O_T group when compared with the group Y (Y), while 818 genes were significantly differently expressed in the O_Y group, and 7057 genes in the T_Y group (Figure 4C and 4D). Moreover, in porcine follicles treated with t-BHP, the common DEGs were enriched in three growth factor signaling pathways, including P53, mTOR, and MAPK, which have been associated with cell proliferation and apoptosis (Figure 4E).

## DISCUSSION

Ovarian failure can lead to infertility, climacterism, or synd. At present, there are various animal models to study female reproductive aging, including gene- mutation models such as FSHR knock-out models [19], and chemical-damage models, such as D-galactose–induced female reproductive senescence model [20]. Although these animal models have shown successful induction of follicular failure and impaired reproductive cycle, the difficulty in obtaining samples from high parity sows and limited research on ovarian senescence warrant an urgent need for an alternative *in vitro* model. Although cellular senescence in other animal models [21] can also lead to multiple secretory phenotypes of senescence, it promotes inflammatory reactions in the body that are not seen during the aging of the ovary; therefore, cellular models cannot simulate the real physiological state of aging of the ovary.

Ovarian aging is the basis of reproductive aging. Female mammals are born with a limited number of primordial follicles. Oxidative stress is a prerequisite for luteinization [22] and disrupts the microenvironment within the follicles [23]. Therefore, this study established an *in vitro* aging model using t-BHP-stimulated follicles, which is simple and easy to operate.

Ovarian function mainly manifests as endocrine function and follicular atresia [24]. E_2_ and P_4_ levels and granulosa cell senescence are classical criteria for ovarian senescence [25]. This study demonstrated that intact antral follicles cultured *in vitro* for up to 36 h resulted in atresia, despite maintaining their morphology. However, within the first 24 h, cell viability was higher and hormone levels of E2 and P4 were normal, indicating that oxidative stress stimulation of follicles within the first 24 h of *in vitro* culture induces senescence. Therefore, this study suggests that *in vitro* culturing of follicles for 24 h with oxidative stress stimulation can be a reliable method to establish an aging model.

The accumulation of oxidative stress has long-term effects on follicular failure [26, 27]. Our preliminary experiments showed that 200 μM is the optimum concentration for oxidative stimulation. Therefore, follicles were cultured with 200 μM t-BHP. The results showed that the ROS level in the treated group was significantly higher than that in the control group, showing a time-dependent increasing trend and impaired granulosa cell function resulting in follicular atresia. Additionally, follicular granulosa cell morphology significantly changed after 6 h of treatment, with SA-β-Gal staining showing that positive granulosa cells were mostly stained after 6 h. These observations indicated that treatment of the follicles with 200 μM t-BHP for 6 h successfully mimicked the ovarian aging phenotype.

The intracellular antioxidant system is involved in various oxidation–reduction reactions in organelles and is activated by a low concentration of ROS. Elevated oxidative stress in cells is the leading cause of cellular [28, 29] and ovarian aging [30]. Mammalian oocytes possess a large number of mitochondria, the organelle where SOD2 is preferentially localized [31]. SOD2 is one of the most important antioxidant enzymes in the body, and it is upregulated following oxidative stress [32, 33]. We observed that oxidative stress significantly upregulated *P53*, *Foxo1*, and *Caspase 3* in the follicles. The *P53* pathway is an important signaling pathway involved in aging [34]. The expression levels of *P53* and *SOD*2 are low in young animal tissues and increase with age. Apoptosis is thought to be activated by the *Caspases 3* pathway, and *Caspases 3* transcriptional regulation is linked to the start of senescence [35]. *Foxo1* induces cellular stress resistance. In our study, western blotting showed that t-BHP upregulated P53, Caspase 3, and Foxo1 and downregulated SOD. The *Foxo1* pathway has been proposed to be an important regulator of apoptosis and senescence [36].

In this study, we analyzed the transcriptomic alterations of three types of porcine follicles and identified shared and specific alterations. Clustering analysis revealed clear separation of young follicles based on their transcriptional signatures, indicating that gene expression abnormalities are a crucial aspect of ovarian aging. Interestingly, we observed that the follicles treated with t-BHP were more similar to normal old follicles samples, and the number of differentially expressed genes between these model samples and normal old samples was lower than that between young samples. We studied the transcription correlation between cultured follicles and naturally aging follicles and proved for the first time that t-BHP treatment (200 μM for 6 h) can successfully simulate *in vivo* aging. We believe that the results of this study have profound implications for the study of ovarian senescence.

In conclusion, we established a porcine follicular senescence model based on t-BHP treatment. t-BHP effectively induced oxidative stress, dysregulation of hormone levels, and follicular damage, causing changes in the expression levels of *SOD*, *P53*, *Caspase 3*, and *Foxo1*. The transcriptomic pattern of the t-BHP–treated cells is similar to that of the naturally aged ovaries. This study lays the foundation for subsequent studies on the decline in reproductive ability caused by reproductive senescence in pigs.

## MATERIALS AND METHODS

### Ovary source

All the experiments were carried out in accordance with the Guidelines for the Protection and Use of Experimental Animals formulated by Animal Protection and Use Committee of Hebei Agricultural University, China. All of the young pigs were 7 to 8 months old. The aging pigs were all breeding sows that had produced more than 7 litters. Ovaries were collected in local slaughterhouse and transported in 37°C saline (containing 100 IU/mL penicillin and 100 μg/mL streptomycin) to our laboratory with within 1 h.

### Culture of intact antral follicles

The ovaries without red/corpus luteum, with uniform and transparent texture and dense distribution of antral follicles, were selected. Antral follicles were removed from the ovaries by using forceps and scalpels. Then the follicles were successively washed with phosphate-buffered saline (PBS) and follicular culture medium. And the diameter of the isolated follicles was measured using a ruler. Follicles of 3–6 mm in diameter with follicular lumen, intact follicular cavity, and abundant blood vessels were selected for culture. The follicles were seeded into twenty-four-well plates containing 7.5% fetal bovine serum (Sigma, USA), 10 mU follicle stimulating hormone (FSH; Ningbo Second Hormone Factory, Zhejiang, China), 1% insulin-transferrin-selenium mixture (ITS; Gibco, USA), 100 μg/mL ascorbic acid (L-AA; Gibco, USA) in Dulbecco’s modified Eagle’s medium/Nutrient Mixture F-12 (DMEM/F12; Hyclone, Logan, UT, USA) and 100 IU/mL penicillin and 100 μg/mL streptomycin, and finally incubated at 37°C with 5% CO_2_. One follicle was randomly placed per well, and 8 follicles were incubated per group. Follicles without drug treatment were cultured in the usual medium and incubated for 0, 24 or 36 h, whereas the treated follicles were maintained in the medium with 200 μM t-BHP. For administration, follicles were incubated for 1, 2, 6 or 12 h. Each treatment was repeated in triplicate.

### ELISA detection of E_2_ and P_4_ in follicle fluid

Following the manufacturer’s instructions, a pig E_2_ ELISA Kit and a swine P_4_ ELISA Kit (both from Yuanmu Biotechnology, Shanghai, China) were used to measure the concentration of E_2_ and P_4_ in the follicular fluid. Fifty microliters of various concentrations of the standard were added into the standard well, and 50 μL of samples to be tested were added into the sample well. Then 100 μL of horseradish peroxidase (HRP) was added to each well and incubated for 60 min at 37°C. Each well was aspirated, and the cells were washed (five washes). Using a pipette, 400 μL of Wash Solution were added to each well. Next, 50 μL each of Chromogen Solutions A and B were added, and the wells were incubated at 37°C for 15 min. Finally, termination solution was added to measure the optical density value at 450 nm.

### Follicular granulosa cell (GC)acquisition

GCs were collected after the follicle culture was completed. The follicles were removed from the twenty-four-well plates with sterile ophthalmic tweezers; the follicle membrane was cut with a sterile surgical blade and follicle fluid was collected. The GCs were seeded into six-well plates containing 15% fetal bovine serum (Sigma, USA) in DMEM/F12 (Hyclone, Logan, UT, USA) and 100 IU/mL penicillin and 100 μg/mL streptomycin, and incubated at 37°C with 5% CO_2_.

### SA-β-Gal staining

The cell senescence β-galactosidase staining package was purchased from Beyotime Biotechnology (C0602, Shanghai, China). GCs of the control group and treatment groups were collected, washed twice with PBS, and fixed with fixing solution at room temperature for 15 min. Then, the cells were washed twice with PBS and incubated overnight with freshly prepared staining solution at 37°C for 12 h, as recommended by the manufacturer. The percentage of SA-β-Gal cells was determined by counting the number of blue cells under bright field illumination and total number of cells in the same field under phase contrast.

### ROS staining

Briefly, DCFH-DA probes were directly added to serum-free medium to be diluted to the working concentration (final concentration: 10 μM), and 1 mL of diluted DCFH-DA solution was added after the medium in the six-well plate was discarded. The medium was incubated for 30 min at 37°C, discarded, and washed with PBS twice before observation under a laser confocal microscope.

### RNA extraction

For the extraction of RNA, we used TRIzol reagent according to the requirements of the manufacturer (TIANGEN, Beijing, China). After mixing fully with the reagent, a single RNA sample was stored in the refrigerator at −80°C for further use. RNA integrity was assessed with Agilent 2100 Bioanalyzer (Agilent Technologies, Santa Clara, CA, USA) and RNA concentration and quality was measured by Spectrophotometer NanoDrop 2000 (Thermo Fisher Scientific, US) (Invitrogen, CA, USA). Total RNA samples that met the following requirements were used: RNA integrity number (RIN) ≥7.0, 28S:18S ratio ≥1.2 and OD260/280 ratio between 1.8 and 2.2.

### qRT-PCR analysis

For qPCR analysis, total RNA (1 ug) was extracted using TRIzol™ reagent according to the manufacturer’s instructions. cDNA was prepared using reverse transcriptase (Ta’KaRa, Dalian, China) and RT Primer Mix. The qPCR reaction was performed using a 7500 F AST Real-Time PCR System (Applied Biosystems, Foster City, CA, USA) according to the 2×Universal Blue SYBR Green qPCR Master Mix (Servicebio, Wuhan, China). The reaction programmed was as follows: pre-denaturation at 95°C for 15 min, then 95°C for 10 s and 60°C for 30 s for 40 amplification cycles. The glyceraldehyde 3-phosphate dehydrogenase (GAPDH) gene was employed as an endogenous control, the relative expression level of genes was calculated with the 2−ΔΔct method. Primers used in this study were synthesized by GENEWIZ (Jiangsu, China) and they are listed in Supplementary Table 1.

### Western blot analysis

Western blot analysis was performed as described previously [37]. Briefly, GCs were collected from the different treatment groups, pelleted by centrifugation (12 000 × g, 5 min), and lysed in RIPA buffer (Solarbio, Beijing, China). Total protein was isolated from GCs by sodium dodecyl sulphate-polyacrylamide gel electrophoresis and subsequently transferred to nitrocellulose membrane. The membranes were blocked with 5% skim milk at room temperature for 2 h, followed by incubation overnight with primary antibodies. The next day, the membranes were washed with PBST and incubated with horseradish peroxidase-conjugated secondary antibodies for 30 min. Protein bands were detected after treatment with SuperSignal West Femto (Thermo Fisher Scientific, Waltham, MA, USA). Primary antibodies against Foxo1 (1:1000), P53 (1:1000), Caspase 3 (1:1000), SOD2 (1:1000) and GAPDH (1:2000) and a secondary antibody conjugated with horseradish peroxidase (HRP; 1:5000) were obtained from Servicebio Biotechnology in Wuhan, China.

### Transcriptome analysis of porcine follicle in correlation and differentially expressed genes

The total RNA of porcine follicles for (three biological replicates) was sequenced using the Illumina NovaSeq 6000 platform (Illumina Inc., San Diego, CA, USA). After filtering out the adapter reads and unqualified reads, clean reads were mapped to the ASR1 reference genome using HISAT2 [38], followed by the assembly and calculation of read count and fragments per kilobase per million values in String Tie [39]. Analysis of differentially expressed genes (DEGs) in each pair of comparisons was performed using the R package DESeq2 [40]. Hierarchical clustering analysis of all transcriptomic samples was performed using the pairwise similarity of each pair of samples determined by the Spearman correlation coefficient. The *P*-value of the differential test was corrected by a multiple hypothesis test, and DEGs were determined by controlling the FDR (false discovery rate) < 0.05. The library construction and transcriptome sequencing were completed by Beijing Annoroad Gene Technology.

### Statistical analysis

Data were presented as mean ± standard error of the mean (SEM) from at least three repeated experiments, as detailed in the Figure legends. Statistical analysis was determined using the SPSS software version 26.0 (SPSS). Differences between multiple groups using one-way analysis of variance (ANOVA) followed by LSD post hoc test. *P* < 0.05 values were considered to be statistically significant. Statistical significance was indicated by different letters. All statistical graphs were drawn by using GraphPad Prism software version 9.0.

### Data accessibility statement

The data supporting the findings in this study are available from the corresponding author upon reasonable request.

## Supplementary Materials

Supplementary Table 1
