# Supplementary material for: Treatment of porcine ovarian follicles with tert-butyl hydroperoxide as an ovarian senescence model in vitro
Source: Aging (Albany NY). 2023 Jul 5;15(13):6212–24. doi: 10.18632/aging.204831 (PMC10373960; doi:10.18632/aging.204831)
Supplement: Supplementary Table 1 [file aging-15-204831-s001.pdf]

SUPPLEMENTARY TABLE

Supplementary Table 1. Primers used in this study.

Gene	Species	Primer sequences (5′–3′)	Annealing temperature (°C)
P53-F	Sus scrofa	GTCACGAACTGGCTGGATG	59
P53-R		GAAGGGACAAAGGACGACAG	
Caspase-3-F	Sus scrofa	GCCATGGTGAAGAAGGAAAA	59
Caspase-3-R		GGCAGGCCTGAATTATGAAA	
SOD-F	Sus scrofa	CATTCCATCATTGGCCGCAC	59
SOD-R		TTACACCACAGGCCAAACGA	
Foxo1-F	Sus scrofa	GCAAATCGAGTTACGGAGGC	59
Foxo1-R		AATGTCATTATGGGGAGGAGAGT	
GAPDH-F	Sus scrofa	GAAGGTCGGAGTGAACGGAT	59
GAPDH-R		CATGGGTAGAATCATACTGGAACA	
